# Mutually Exclusive Uncertainty Relations

**DOI:** 10.1038/srep36616

**Published:** 2016-11-08

**Authors:** Yunlong Xiao, Naihuan Jing

**Affiliations:** 1 School of Mathematics, South China University of Technology, Guangzhou 510640, China; 2 Max Planck Institute for Mathematics in the Sciences, Leipzig 04103, Germany; 3 Department of Mathematics, North Carolina State University, Raleigh, NC 27695, USA

## Abstract

The uncertainty principle is one of the characteristic properties of quantum theory based on incompatibility. Apart from the incompatible relation of quantum states, mutually exclusiveness is another remarkable phenomenon in the information- theoretic foundation of quantum theory. We investigate the role of mutual exclusive physical states in the recent work of stronger uncertainty relations for all incompatible observables by Mccone and Pati and generalize the weighted uncertainty relation to the product form as well as their multi-observable analogues. The new bounds capture both incompatibility and mutually exclusiveness, and are tighter compared with the existing bounds.

Heisenberg’s uncertainty principle^1^ is one of the fundamental notions in quantum theory. The original form is a result of noncommutativity of the position and momentum operators. Robertson’s formulation of Heisenberg’s uncertainty principle in matrix mechanics^2^ states that for any pair of observables *A* and *B* with bounded spectrums, the product of standard deviations of *A* and *B* is no less than half of the modulus of the expectation value of their commutator:


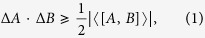


where 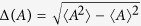 is the standard deviation of the self-adjoint operator *A*. Here the expectation value 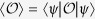 is over the state 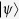 for any observable 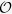. In fact, Robertson’s uncertainty relation can be derived from a slightly strengthened Schrödinger uncertainty inequality^3^


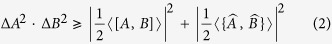


where 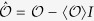.

Besides their importance in quantum mechanics, uncertainty relations play a significant role in quantum information theory as well^4^,^5^,^6^,^7^,^8^,^9^,^10^. The variance-based uncertainty relations possess clear physical meanings and have a variety of applications in quantum information processings such as quantum spin squeezing^11^,^12^,^13^,^14^,^15^, quantum metrology^16^,^17^,^18^, and quantum nonlocality^19^,^20^.

While the early forms of variance-based uncertainty relations are vital to the foundation of quantum theory, there are two problems still need to be addressed: (1) Homogeneous product of variances may not fully capture the concept of incompatibility. In other words, a weighted relation may produce a better approximation (e.g., the uncertainty relation with Rényi entropy^21^ and variance-based uncertainty relation for a weighted sum), for more details and examples, see ref. 22; (2) The existing variance-based uncertainty relations are far from being tight, and improvement is needed. One also needs to know how to generalize the product form to the case of multiple observables for practical applications.

In ref. 22, the authors and collaborators have proposed weighted uncertainty relations to answer the first question and succeeded in improving the uncertainty relation. Let’s recall the weighted uncertainty relation for the sum of variances. For arbitrary two incompatible observables *A, B* and any real number *λ*, the following inequality holds


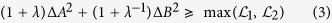


with


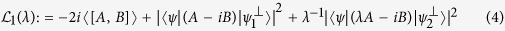


and


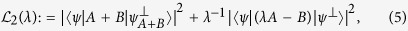


where 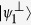, 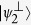, 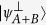 and 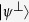 are orthogonal to |*ψ*〉. In information-theoretic context, it is also natural to quantify the uncertainty by weighted products of variances, which also help to estimate individual variance as in ref. 22.

Recently, Maccone and Pati obtained an amended Heisenberg-Robertson inequality^23^:


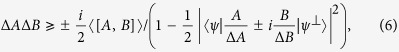


which is reduced to Heisenberg-Robertson’s uncertainty relation when minimizing the lower bound over 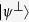, and the equality holds at the maximum. This amended inequality gives rise to a stronger uncertainty relation for almost all incompatible observables, and the improvement is due to the special vector 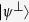 perpendicular to the quantum state |*ψ*〉. We notice that this can be further improved by using the mutually exclusive relation between 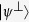 and |*ψ*〉. Moreover, this idea can be generalized to the case of multi-observables. For this reason the strengthen uncertainty relation thus obtained will be called a *mutually exclusive uncertainty relation*.

The goal of this paper is to answer the aforementioned questions to derive the product form of the weighted uncertainty relation, and investigate the physical meaning and applications of the mutual exclusive physical states in variance-based uncertainty relations. Moreover, we will generalize the product form to multi-observables to give tighter lower bounds.

## Results

We first generalize the weighted uncertainty relations from the sum form^22^ to the product form, and then introduce *mutually exclusive uncertainty relations* (MEUR). After that we derive a couple of lower bounds based on *Mutually exclusive physical states* (MEPS), and we show that our results outperform the bound in ref. 23, which has been experimentally tested recently^24^. Finally, generalization to multi-observables is also given.

We start with the sum form of the uncertainty relation, which takes equal contribution of the variance from each observable. However, almost all variance-based uncertainty relations do not work for the general situation of incompatible observables, and they often exclude important cases. In ref. 22, the authors and collaborators solved this degeneracy problem by considering weighted uncertainty relations to measure the uncertainty in all cases of incompatible observables. Using the same idea, we will study the product form of weighted uncertainty relations to give new and alternative uncertainty relations in the general situation. The corresponding mathematical tool is the famous *Young’s inequality*. The new weighted uncertainty is expected to reveal the lopsided influence from observables. They contain the usual homogeneous relation of Δ*A*^2^Δ*B*^2^ as a special case.

**Theorem 1.** *Let A, B be two observables such that* Δ*A*Δ*B* > 0*, and p, q two real numbers such that* 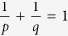
*. Then the following weighted uncertainty relation for the product of variances holds*.


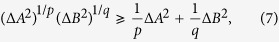


*where p* < 1, *and the equality holds if and only if* Δ*A* = Δ*B. If p* > 1, *then* 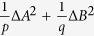 *becomes a upper bound for the weighted product*.

See Methods for a proof of Theorem 1.

The weighted uncertainty relations for the product of variances have a desirable feature: our measurement of incompatibility is weighted, which fits well with the reality that observables usually don’t always reach equilibrium, i.e., in physical experiments their contributions may not be the same (cf. ref. 22). As an illustration, let us consider the *relative error function* between the uncertainty and weighted bound, which is defined by


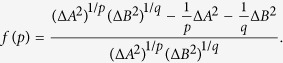


In general *f* is a function of both *p* and |*ψ*〉. It is hard to find its extremal points as it involves in partial differential equations. Also the extremal points hardly occur at homogeneous weights, so incompatible observables usually don’t contribute equally to the uncertainty relation, which explains the need for a weighted uncertainty relation in the product form.

In what follows, we show how to tighten Maccone and Pati’s amended Heisenberg-Robertson uncertainty relation^23^ by regarding mutually exclusive physical states as another information resource, and then generalize the variance-based uncertainty relation to the case of multi-observables.

We will refer to (6) as a *mutually exclusive uncertainty relation* since the states |*ψ*〉 and 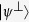 represent two mutual exclusive states in quantum mechanics, which is the main reason for improving the tightness of the bound. Next we move further to improve the bound by combining mutually exclusive relations and weighted relations.

Maccone and Pati’s uncertainty relation can be viewed as a singular case in a family of uncertainty relations parameterized by positive variable *λ*, which corresponds to our recent work on weighted sum of uncertainty relations^22^. We proceed similarly as the case of the amended Heisenberg-Robertson uncertainty relation by considering a modified square-modulus and Holevo inequalities in Hilbert space^25^ in the following result.

**Theorem 2.** *Let A and B be two incompatible observables and* |*ψ*〉 *a fixed quantum state. Then the mutually exclusive uncertainty relation holds:*


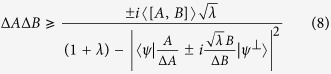


*for any unit vector* 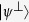 *perpendicular to* |*ψ*〉 *and arbitrary parameter λ* > 0.

See Methods for a proof of Theorem 2.

The obtained variance-based uncertainty relation is stronger than Maccone and Pati’s amended uncertainty relation. In fact, when the maximal value 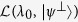 is reached at a point *λ*_0_ ≠ 1, the new bound is stronger than that of Maccone-Pati’s amended uncertainty relation. Let 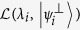 (*i* = 1, 2) be two lower bounds given in the RHS of (8), define the *tropical sum*


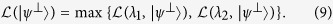


This gives a tighter lower bound when the maximal value of 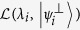 is reached at different direction in *H*_*ψ*_ (hyperplane orthogonal to |*ψ*〉) for 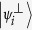. In other words, the new lower bound is a piecewise defined function of MEPS 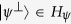 taking the maximum of the two bounds. In particular, for *λ*_0_ ≠ 1, the tropical sum 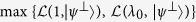 offers a better lower bound than 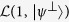, the Maccone-Pati’s lower bound. Note that 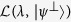 may have a smaller minimum value than 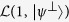 when *λ* ≠ 1, as 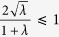, while the minimum value of 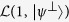 is just the bound for Heisenberg-Robertson’s uncertainty relation. Because we only consider the maximum, it does not affect our result.

For example, consider a 4-dimensional system with state 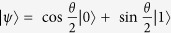, 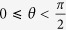 and take the following observables


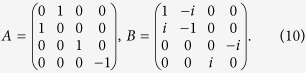


Direct calculation gives


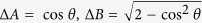


and


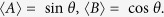


For 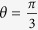 and 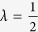, set


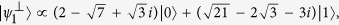


and


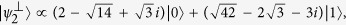


both of them have modulus one, then


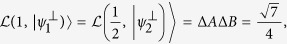


meanwhile


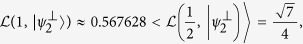


so


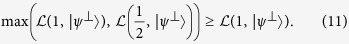


**Figure 1:**
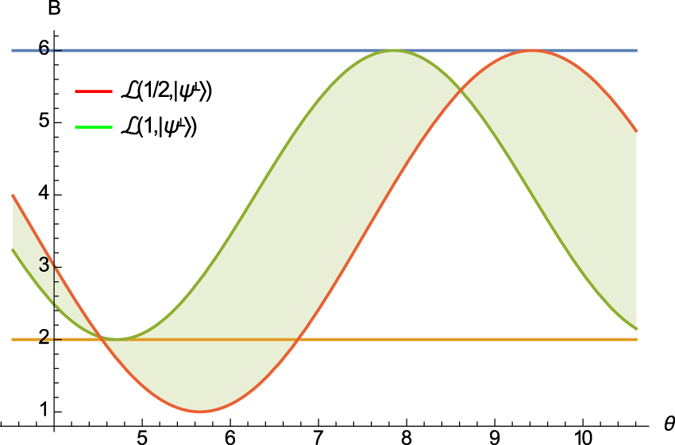
Schematic comparison of bounds: Top and middle lines are Δ*A*Δ*B* and 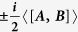 resp. Tropical sum 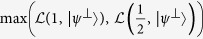 is the upper boundary above the shadow, and the red and green ones are 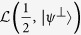 and Maccone-Pati’s bound 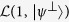 resp.

**Figure 2:**
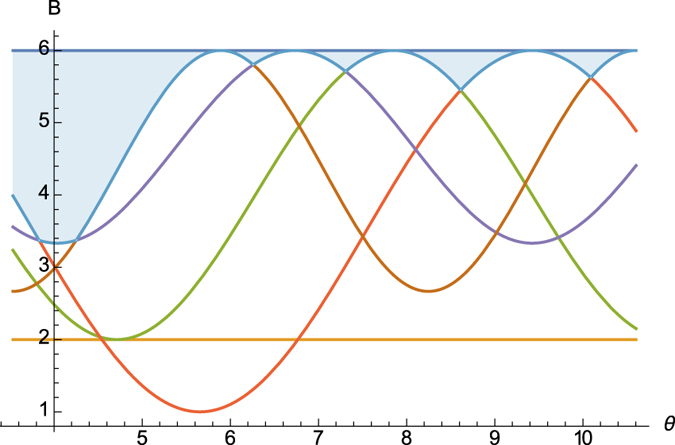
Schematic comparison: Top line is Δ*A*Δ*B*. Blue curve is our bound 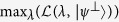, the shadow region is the difference between Δ*A*Δ*B* and our bound 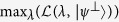. Other bounds are shown in different colors.

Both the lower bounds 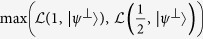 and 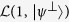 are functions of MEPS 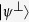. However, for each 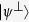, 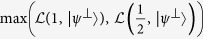 gives a better approximation of Δ*A*Δ*B* than 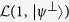. Figure 1 is a schematic diagram of these two lower bounds. It is clear that 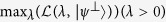 provides a closer estimate to Δ*A*Δ*B*:


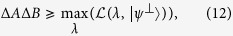


for any unit MEPS 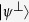 orthogonal to |*ψ*〉. This is due to the fact that the bound 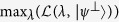 is continuous on both MEPS 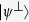 and *λ*, which shows the advantage of our mutually exclusive uncertainty principle. The shadow region in Fig. 2. illustrates the outline of Δ*A*Δ*B* and our bound 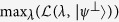.

**Figure 3:**
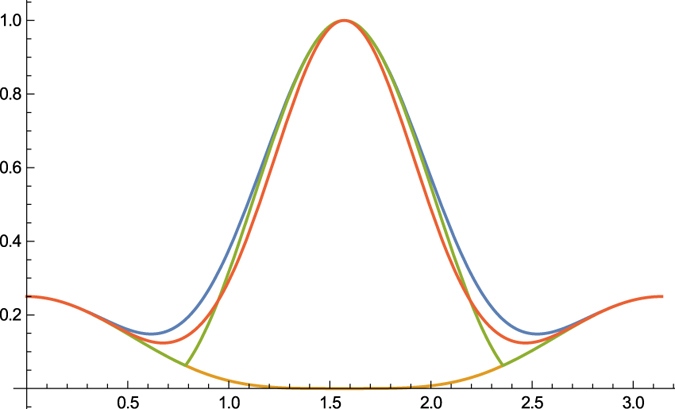
Lower bounds of Δ*A*^2^Δ*B*^2^ for a family of spin-1 particles. Δ*A*^2^Δ*B*^2^, our bound 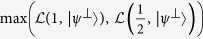, the bound given by Eq. (3) in ref. 26 and the Schrödinger bound are respectively shown in blue, orange, green and yellow.

In Fig. 3, we illustrate our results, showing how the obtained bound 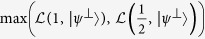 outperforms the recent work of ref. 26 as well as the Schrödinger uncertainty relation. We consider the angular momenta *L*_*x*_ and *L*_*y*_ for spin-1 particle with state |*ψ*〉 = cos *θ* |1〉 − sin *θ* |0〉 and 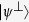 = sin *θ* |1〉 + cos *θ* |0〉, where |0〉 and |1〉 are eigenstates of the angular momentum *L*_*z*_.

Mutually exclusive physical states with different directions in *H*_*ψ*_ offer different kinds of mutually exclusive information and improvement of the uncertainty relation. When such an experiment of the mutually exclusive uncertainty relation is performed, one is expected to have infinitely many strong lower bounds of the variance-based uncertainty relation.

Now we further generalize the uncertainty relations to multi-observables. For simplicity, write


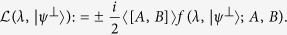


So


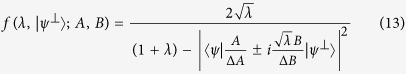


is continuous on both MEPS 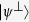 and *λ*. Repeatedly using (8) for 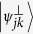 and *λ*_*jk*_, we obtain the following relation.

**Theorem 3.** *Let A*_1_*, A*_2_, …*, A*_*n*_ *be n incompatible observables,* |*ψ*〉 *a fixed quantum state and λ*_*jk*_ *positive real numbers, we have*


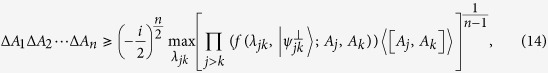


*for any MEPS* 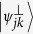 *orthogonal to* |*ψ*〉 *with modulus one. If some* 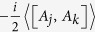 *is negative, a negative sign is inserted into the RHS of* (14) *to ensure positivity. The equality holds if and only if MEPS* 
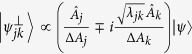
 *for all j* > *k*.

As a corollary, Theorem 3 leads to a simple bound of the uncertainty relation for multi-observables.

**Corollary 1.** *Let A*_1_*, A*_2_, …, *A*_*n*_ *be n incompatible observables, then the following uncertainty relation holds*


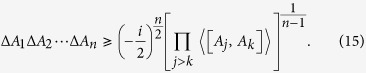


See Methods for a proof of Corollary 1.

Next, we provide yet another mutually exclusive uncertainty relation.

**Theorem 4.** *Let A and B be two incompatible observables and* |*ψ*〉 *a fixed quantum state. Then*


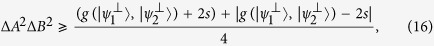


*for any unit MEPS* 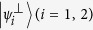 *orthogonal to* |*ψ*〉, *with*


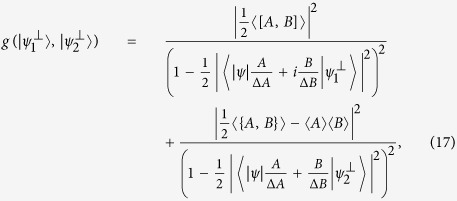


*where MEPS* 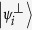 *are unit vectors in H*_*ψ*_.

See Methods for a proof of Theorem 4.

**Figure 4:**
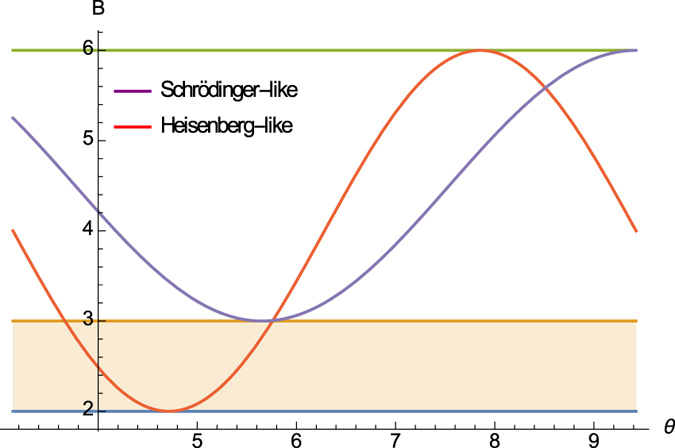
Schematic comparison of uncertainty relations. Top, middle and bottom lines are Δ*A*^2^Δ*B*^2^, Schrödinger’s and the square of Heisenberg’s bounds resp. Orange and purple curves are the square of Maccone-Pati’s amended Heisenberg bound and our amended Schrödinger bound resp.

Obviously, (16) can be seen as an *amended Schrödinger inequality* and also offers a better bound than (2) and Maccone-Pati’s relation (6). Figure 4 illustrates the schematic comparison.

In general, if there exists an operator *M* for *A* and *B* such that 〈*M*〉 = 0, 
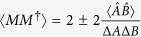
, then we have the following:

**Remark 1.** *Let A and B be two incompatible observables and* |*ψ*〉 *a fixed quantum state. We claim the following mutually exclusive uncertainty relation holds:*


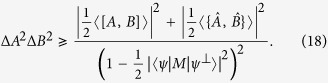


Eq. (18) also gives a generalized Schrödinger uncertainty relation. Here as usual MEPS 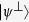 is any unit vector perpendicular to |*ψ*〉. The proof of Theorem 4 and Remark 1 are similar to that of Theorem 2, so we sketch it here. It is easy to see that the RHS of (18) reduces to the lower bound of Schrödinger’s uncertainty relation (2) when minimizing over 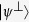, and the equality holds at the maximum. The corresponding uncertainty relation for arbitrary *n* observables is the following result.

**Theorem 5.** *Let A*_1_*, A*_2_, …*, A*_*n*_ *be n incompatible observables,* |*ψ*〉 *a fixed quantum state and λ*_*jk*_ *positive real numbers. Then we have that*


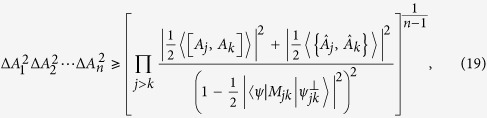


*where M*_*jk*_ *satisfy* 〈*M*_*jk*_〉 = 0, 
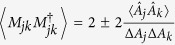
 *and MEPS* 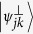 *orthogonal to* |*ψ*〉 *with modulus one*.

The RHS of (19) has the minimum value


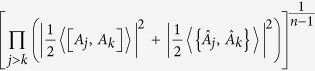


and the equality holds at the maximum. Therefore one obtains the following corollary.

**Corollary 2.** *Let A*_1_*, A*_2_, …*, A*_*n*_ *be n incompatible observables, then the following uncertainty relation holds*


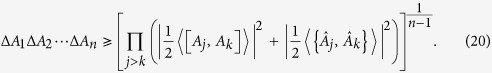


See Methods for a proof of Corollary 2.

We note that our enhanced Schrödinger uncertainty relations offer significantly tighter lower bounds than that of Maccone-Pati’s uncertainty relations for multi-observables, as our lower bound contains an extra term of 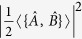 (compare (1) with (2)).

Finally, we remark that we can also replace the non-hermitian operator 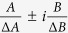 in (6) by a hermitian one. A natural consideration is the amended uncertainty relation


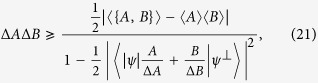


for any unit MEPS 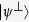 perpendicular to |*ψ*〉. The corresponding uncertainty relation for multi-observables can also be generalized.

The minimum of Maccone and Pati’s amended bound 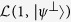 in the RHS of (6) agrees with the bound in Heisenberg-Robertson’s uncertainty relation, which is weaker than Schrödinger’s bound in (2). We point out that the bound given as a continuous function of MEPS’s will always produce a better lower bound. In fact, the continuity of 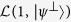 in MEPS 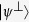 implies that there exists suitable 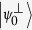 such that 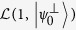 is tighter than the bound of Heisenberg-Robertson’s uncertainty relation. Similarly our lower bound given in (27) or more generally in (18) provides a tighter lower bound than the enhanced Schrödinger’s uncertainty relation (2). This shows the advantage of lower bounds with MEPS’s. Furthermore, lower bounds with more variables give better estimates for the product of variances of observables, as in (19).

## Conclusions

The Heisenberg-Robertson uncertainty relation is a fundamental principle of quantum theory. It has been recently generalized by Maccone and Pati to an enhanced uncertainty relation for two observables via mutually exclusive physical states. Based on these and weighted uncertainty relations^22^, we have derived uncertainty relations for the product of variances from mutually exclusive physical states (MEPS) and offered tighter bounds.

In summary, we have proposed generalization of variance-based uncertainty relations. By virtue of MEPS, we have introduced a family of infinitely many Schrödinger-like uncertainty relations with tighter lower bounds for the product of variances. Indeed, our mutually exclusive uncertainty relations can be degenerated to the classical variance-based uncertainty relations by fixing MEPS and the weight. Also, our study further shows that the mutually exclusiveness between states is a promising information resource.

## Methods

**Proof of Theorem 1.** To prove the theorem, we recall *Young’s inequality*^27^: for 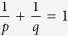, *p* < 1 one has that


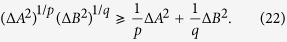


Note that the right-hand side (RHS) may be negative if *p* < 1. But this can be avoided by using the symmetry of Young’s inequality to get


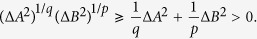


Thus our bound is nontrivial. We remark that if *p* > 1, it is directly from the *Young’s inequality*^27^


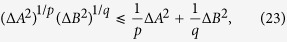


and equality holds in (22) and (23) only when Δ*A* = Δ*B*.                      ■

**Proof of Theorem 2.** Here we provide two proofs of the proposed mutually exclusive uncertainty relation (8). The first one, based on weighted relations^22^, is a natural deformation of ref. 23 and is sketched as follows. By maximizing the RHS of (8), we see that the maximum Δ*A*Δ*B* is achieved when the *mutually exclusive physical state* (MEPS) 
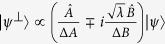
. Clearly our uncertainty relation contains (6) as a special case of *λ* = 1.

The second proof uses geometric property and is preferred because of its mathematical simplicity and also working for the amended Heisenberg-Robertson uncertainty relation^23^. In fact, the RHS of (6), denoted by 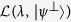, is a continuous function of *λ* and the unit MEPS 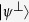. By the vector projection, the maximum value Δ*A*Δ*B* of 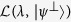 over the hyperplane of 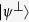 is attained when 
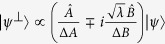
. Therefore for any *λ* > 0


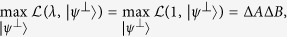


where 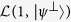 is the RHS of (6). Similarly


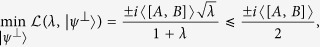


for any *λ* > 0 and the equality holds if *λ* = 1, which implies (8) and completes the second proof.       ■

**Proof of Corollary 1.** Obviously, taking the minimum of (14) over MEPS 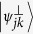 implies that


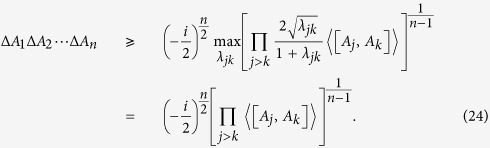


When *λ*_*jk*_ = 1 for all *j* > *k*, the minimum is 
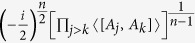
. Meanwhile if *λ*_*jk*_ and MEPS 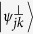 vary, Eq. (14) provides a family of mutually exclusive uncertainty relations for arbitrary *n* observables with (24) as the lower bound.               ■

**Proof of Theorem 4.** By the same method used in deriving (8) it follows that 
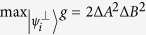
, and 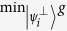 is


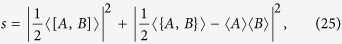


which equals to the lower bound of the Schrödinger uncertainty (2). We can modify *g* into a function with the same maximum and lower bound as Schrödinger’s uncertainty relation. Note that *s* ≤ Δ*A*^2^Δ*B*^2^, then


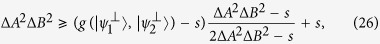


which is equivalent to (by solving Δ*A*^2^Δ*B*^2^)


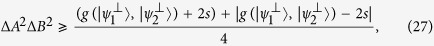


for any unit MEPS 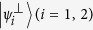 orthogonal to |*ψ*〉. In fact, let 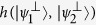 be the RHS of (27). It is easy to see that 
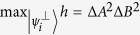
 and


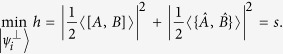


Hence we have the mutually exclusive uncertainty relation appeared in (27).            ■

**Proof of Corollary 2.** Apparently, taking the minimum of (19) over MEPS 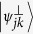 implies that


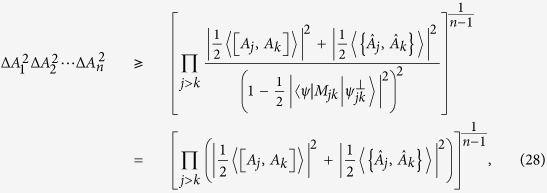


with the minimum is 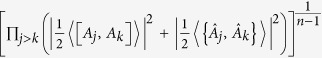. Meanwhile if the MEPS 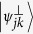 vary, Eq. (19) provides a family of mutually exclusive uncertainty relations for arbitrary *n* observables with (28) as the lower bound.               ■

## Additional Information

**How to cite this article**: Xiao, Y. and Jing, N. Mutually Exclusive Uncertainty Relations. *Sci. Rep.* **6**, 36616; doi: 10.1038/srep36616 (2016).

**Publisher’s note:** Springer Nature remains neutral with regard to jurisdictional claims in published maps and institutional affiliations.
